# Cryptic HBV Replicative Activity Is Frequently Revealed in Anti-HBc-Positive/HBsAg-Negative Patients with HIV Infection by Highly Sensitive Molecular Assays, and Can Be Predicted by Integrating Classical and Novel Serological HBV Markers

**DOI:** 10.3390/microorganisms8111819

**Published:** 2020-11-18

**Authors:** Romina Salpini, Vincenzo Malagnino, Lorenzo Piermatteo, Tiziana Mulas, Mohammad Alkhatib, Rossana Scutari, Elisabetta Teti, Carlotta Cerva, Katia Yu La Rosa, Marta Brugneti, Ada Bertoli, Benedetta Rossi, Vera Holzmayer, Jeffrey Gersch, Mary Kuhns, Gavin Cloherty, Francesca Ceccherini-Silberstein, Carlo-Federico Perno, Marco Iannetta, Massimo Andreoni, Loredana Sarmati, Valentina Svicher

**Affiliations:** 1 Department of Experimental Medicine, University of Rome Tor Vergata, 00133 Rome, Italy; rsalpini@gmail.com (R.S.); piermatteolorenzo@gmail.com (L.P.); mohammad--alkhatib@hotmail.com (M.A.); scutari.rossana@gmail.com (R.S.); y.katia@hotmail.it (K.Y.L.R.); marta.b88@hotmail.it (M.B.); bertoli@uniroma2.it (A.B.); ceccherini@med.uniroma2.it (F.C.-S.); 2 Clinic of Infectious Diseases, Tor Vergata University Hospital, 00133 Rome, Italy; malagninovincenzo@gmail.com (V.M.); tiziamulas@gmail.com (T.M.); elisabetta.teti@gmail.com (E.T.); carlottacerva@gmail.com (C.C.); benedetta.rossi19@gmail.com (B.R.); marco.iannetta@uniroma2.it (M.I.); andreoni@uniroma2.it (M.A.); sarmati@uniroma2.it (L.S.); 3 Abbott Molecular, Des Plaines, IL 60018-3315, USA; Vera.Holzmayer@abbott.com (V.H.); jeffrey.gersch@abbott.com (J.G.); mary.kuhns@abbott.com (M.K.); gavin.cloherty@abbott.com (G.C.); 4 Microbiology and Immunology Diagnostics, Ospedale Pediatrico Bambino Gesù, 00165 Rome, Italy; cf.perno@uniroma2.it

**Keywords:** HBV, HIV, anti-HBc titer, anti-HBs titer, serum HBV-DNA, serum HBV-RNA, droplet digital PCR

## Abstract

The anti-HBc-positive/HBsAg-negative status is frequent in HIV-infection and correlates with poor survival. Here, by highly-sensitive assays, we evaluate cryptic HBV replication and factors correlated with its detection in 81 anti-HBc-positive/HBsAg-negative HIV-infected patients. Patients were treated for >12 months with HBV-active modern combined antiretroviral-therapy (cART) and had serum HBV-DNA < 20 IU/mL by commercial Real-Time PCR. Serum HBV-DNA was quantified by droplet digital PCR, serum HBV-RNA by an Abbott research assay, and anti-HBc titer (proposed to infer intrahepatic cccDNA) by Lumipulse/Fujirebio. Cryptic serum HBV-DNA was detected in 29.6% of patients (median (IQR): 4(1–15) IU/mL) and serum HBV-RNA in 3.7% of patients despite HBsAg-negativity and HBV-active cART. Notably, cryptic serum HBV-DNA correlated with an advanced CDC-stage (*p* = 0.01) and a lower anti-HBs titer (*p* = 0.05), while serum HBV-RNA correlated with lower nadir CD4+ cell-count (*p* = 0.01). By analyzing serological HBV-markers, the combination of anti-HBs < 50 mIU/mL (indicating lower immune response) plus anti-HBc > 15COI (reflecting higher HBV replicative activity) was predictive of cryptic serum HBV-DNA (OR: 4.7(1.1–21.7), *p* = 0.046, PPV = 62.5%, and NPV = 72%). In conclusion, cryptic HBV-replication (not detected by classical assays) characterizes a conspicuous set of anti-HBc-positive HIV-infected patients despite HBsAg-negativity and HBV-active combined antiretroviral therapy (cART). The integration of classical and novel markers may help identify patients with cryptic HBV-replication, thus optimizing the monitoring of anti-HBc-positive/HBsAg-negative HIV-infected patients.

## 1. Introduction

Worldwide, it has been estimated that 7.4% of HIV-infected patients harbour a chronic HBV infection, corresponding to 2.7 million individuals [1]. This percentage undergoes a remarkable increase considering HIV-infected patients with HBsAg negativity/anti-HBc positivity, which is a serological profile that could hide an occult hepatitis B infection. In particular, 15–30% of HIV-infected patients are anti-HBc-positive/HBsAg-negative, reaching peaks of 80% in geographic areas endemic for HBV infection [2,3]. This issue is particularly relevant considering that the anti-HBc-positivity/HBsAg-negativity status in HIV-positive people has been associated with an increased risk of progression to liver diseases [4], a higher mortality related to AIDS events [5], and a poor control of HIV replication during combined antiretroviral therapy (cART) [6]. Nevertheless, so far, no clear suggestions are provided regarding the management of HIV-positive anti-HBc-positive/HBsAg-negative patients [7].

In anti-HBc-positive/HBsAg-negative patients, serum HBV-DNA can be present at very low concentrations, rarely detected by the currently available commercial HBV-DNA assays. In the setting of HIV-infection, according to previous studies (mainly led in highly endemic countries for HBV infection), the percentage of anti-HBc-positive/HBsAg-negative patients with cryptic serum HBV-DNA has been reported to range from 4% to 24% [8,9,10,11]. However, the frequency of cryptic HBV viremia in this setting of patients remains controversial mainly owing to differences in sensitivity and specificity among the detection methods used to detect serum HBV-DNA in the different studies. In this light, it is particularly relevant to apply highly sensitive molecular assays, capable of detecting few viral copies in the setting of anti-HBc/HBsAg-negative patients [12]. Understanding this issue is critical since cryptic HBV-replication may represent a factor fueling liver disease progression [13,14,15] and exposing patients to a higher risk of viral reactivation in the setting of an exacerbated immune-compromised status [16]. 

Recently, novel biomarkers have been proposed to reflect the intrahepatic HBV reservoir [17,18], providing information on the clinical evolution and therapeutic outcome of HBV infection. Among them, serum HBV-RNA measures the amount of viral particles containing pre-genomic RNA molecules lacking the reverse transcriptase process [19]. This biomarker has been proposed as a minimally invasive method to track the transcriptional activity of the covalently closed circular DNA (cccDNA) [20]. Another recent biomarker is represented by the titers of anti-HBc. In HBsAg-positive patients, anti-HBc titers vary among the different phases of chronic infection [21,22], and are associated with inflammatory activity and significant fibrosis in drug-naive patients [23,24]. Furthermore, in anti-HBc-positive/HBsAg-negative individuals, an anti-HBc titer above the threshold of 4 cut-off index (COI) can predict the presence of intrahepatic cccDNA [25]. However, the role of these HBV markers has been mainly investigated in chronically HBV mono-infected patients, while there is still a paucity of information regarding their potential usefulness in the setting of HIV coinfection.

In this light, this study is aimed at (i) investigating cryptic HBV replicative activity (in term of serum HBV-DNA and serum HBV-RNA) by recently-proposed and highly sensitive molecular assays, (ii) deciphering factors correlated with its detection, (iii) defining the role of serological markers (anti-HBc and anti-HBs titers) in predicting cryptic HBV replicative activity in a well-characterized set of anti-HBc-positive/HBsAg-negative HIV-infected patients receiving an HBV-active cART.

## 2. Materials and Methods

### 2.1. Study Design

This study was conducted at the Infectious Diseases clinic of the Policlinico “Tor Vergata” in Rome on 81 HIV-positive patients, who are all anti-HBc-positive and HBsAg-negative. All patients were treated with a cART containing >1 anti-HBV drug at the time of sample collection.

The following information was collected from all the included patients: sex, contagion risk factors, country of origin, date of HIV diagnosis, quantitative HIV-RNA (copies/mL) and CD4 + T cell count (cells/uL) at HIV diagnosis and at blood sample collection, cART start date, virological success date, defined as the detection of two consecutive HIV-RNA values <50 copies/mL, virological failure date defined as the detection of two consecutive HIV-RNA values >50 copies/mL or a single detection >1000 copies/mL, HCV coinfection, CDC stage, and levels of liver transaminases.

At blood sample collection, the routine blood analysis and the complete panel of the classical serological markers for HBV (HBsAg, HBeAg, anti-HBc, anti-HBe, and anti-HBs), and serum HBV-DNA levels were assessed for all the subjects included in the study. Furthermore, for all patients, the following parameters were quantified: (i) serum HBV-DNA by using an in-house highly sensitive droplet digital PCR (ddPCR) assay, (ii) serum HBV-RNA, and (iii) the titers of the anti-HBc antibodies. 

### 2.2. Quantification of Classical HBV Markers

Serum HBsAg was tested by using the commercially available quantitative HBsAg detection system (Abbott Architect, lower limit of detection: 50 mIU/mL, routinely used at Tor Vergata Policlinic for diagnostic purposes) (Abbott Diagnostics, Abbott Park, IL, USA). Similarly, the qualitative detection of HBeAg, anti-HBe, and anti-HBs serological markers was performed by using the Abbott Architect instrument (Abbott Diagnostics, Abbott Park, IL, USA).

Serum HBV-DNA was quantified by a fully automated Real-Time PCR system (COBAS Amplicor-COBAS TaqMan, Roche, Basel, Switzerland), characterized by a lower limit of quantitation (LLoQ) of 20 IU/mL.

### 2.3. Highly Sensitive Detection of HBsAg

HBsAg was also detected by the highly sensitive Abbott ARCHITECT HBsAg NEXT Qualitative assay (HBsAgNx) with a sensitivity of 0.005 IU/ml (Abbott Diagnostics, Abbott Park, IL, USA).

### 2.4. Quantification of Serum HBV-DNA by Highly Sensitive-ddPCR

Serum HBV-DNA was also quantified by using an in-house highly sensitive assay based on the ddPCR technology with a lower limit of quantification of 1 IU/mL, and a high reproducibility and linearity within the range of HBV-DNA from 1 to 10,000 IU/mL. This assay targets a 102 nt sequence of the S gene region HBV-DNA by using the following primers and probe: Forward primer: 5′-TTATCGCTGGATGTGTCT-3′; Reverse primer: 5′-CAAGAAGATGAGGCATAGC-3′ and Probe: Hex-5′-AGAGGAAgATGATAAAACGC-3′. ddPCR assay is performed using all DNA extracted from 2 mL of patient’s plasma. Briefly, serum HBV-DNA was extracted twice from 1 mL of serum with the Nucleic Acid Extraction System eMAG (Biomerieux, Marcy-l’Étoile, France) extractor in 50 µL of elution buffer in duplicate. Both extracts (thus, deriving from 2 mL of plasma) were combined, dried, and concentrated in 20 µL of nuclease-free water. For ddPCR quantifications, 5 µL of DNA extract was added to 15 µL of PCR reaction in quadruplicate, in order to detect any HBV-DNA copy, possibly present in the 2 mL of plasma. 

The procedure for ddPCR was as follows: a 20 μL of ddPCR reaction mix was prepared with 10 μL of 2× ddPCR Supermix for probes (no dUTP) (Bio-Rad, Pleasanton, CA, USA), 1 μL of 20× primers/probe mix, 4 μL of nuclease-free water, and 5 μL of extracted DNA. Reaction droplets were generated according to manufacturer’s instructions by using the QX200TM Droplet Generator (Bio-Rad, Hercules, CA, USA). Then, HBV-DNA was amplified using T100TM Thermal Cycler (Bio-Rad, Hercules, CA, USA). After amplification, positive droplets were quantified by the QX200TM Droplet Reader (Bio-Rad, Hercules, CA, USA) and analysed by using the QuantaSoftTM software (Bio-Rad, Hercules, CA, USA). At least three negative controls and one positive control was included in each ddPCR reaction to verify the efficiency of amplification and to exclude the samples’ contamination.

### 2.5. Quantification of Serum HBV-RNA by the Abbott Research Assay

The levels of serum pregenomic HBV-RNA were assessed by using the Abbott Real-Time HBV RNA Research Use Only assay (Abbott Diagnostics, Abbott Park, IL, USA), as described in Butler et al., 2018 [26]. Results are expressed as log10 U/mL. The lower limit of quantitation of the assay is 1.65 log U/mL.

### 2.6. Quantification of Antibodies Anti-HBc

The levels of anti-HBc were quantified by using the Lumipulse^®^ G HBcAb-N assay (Fujirebio, Tokyo, Japan). The assay is based on a fully automated two-step sandwich CLEIA technology working on LUMIPULSE^®^ G system. Anti-HBc IgG levels are automatically reported as COI, calculated as a multiple of the cut-off value obtained from calibration data (COI = S/C × 0.09). The lower limit of quantification reported by the manufacturer for the assay is 1 COI.

### 2.7. Ethics Committee

An ethics committee’s consent to the study was obtained on the 6 November 2018 by the Fondazione PTV Policlinico Tor Vergata (Code: RS.178.18). As far as privacy is concerned, all personal information was treated confidentially and all clinical data were analyzed anonymously. The biological samples were kept in the Virology Laboratory of the University of Rome Tor Vergata. Only the staff involved in the study had access to biological samples. 

### 2.8. Statistical Methods

Mann-Whitney test for continuous variables and Chi-Squared test for discrete variables were applied to define statistically significant differences. Correlations between virological parameters were assessed by the Spearman Test. The area under the receiver operating characteristics (AUROC) was used to define the thresholds of anti-HBc and anti-HBs with the best performance in predicting cryptic serum HBV-DNA.

## 3. Results

### 3.1. Study Population Description

This study included 81 consecutive HIV-infected patients positive for anti-HBc and negative for HBsAg receiving a cART containing > 1 anti-HBV drug for at least 12 months at blood sample collection (median (IQR) duration of treatment: 35 (17–61) months) (Table 1). Most patients were male (71.6%) with a median (IQR) age of 55 (47–63) years. At blood sample collection, 75% of patients have a plasma HIV-RNA < 20 copies/mL (indicative of virological success), while the remaining 25% experienced a virological rebound with a median (IQR) plasma HIV-RNA of 69 (42–792) copies/mL. At blood sample collection, CD4+ cell count was 541 (349–716) cells/l (Table 1). 

Thirty-one (38.8%) patients have been diagnosed in CDC stage A while 21 (26.2%) in B and 28 (35.0%) in C stage, respectively (Table 1).

At the blood sample collection, all anti-HBc-positive/HBsAg-negative HIV-infected patients had a serum HBV-DNA < 20 IU/mL by the commercially available Real-Time PCR. Their median anti-HBc titer was 4.2 (2.4–11.6) COI (Table 2). Overall, 71.6% of patients were also positive to anti-HBs with a median (IQR) titer of 277 (90–957) mIU/mL (Table 1). The highly sensitive detection of HBsAg confirmed the negativity for this marker in all patients (Table 1).

Regarding cART, all patients received drugs active against HBV: 37 (45.7%) tenofovir alafenamide/emtricitabine (TAF/FTC), 25 (30.9%) tenofovir disoproxil fumarate/emtricitabine (TDF/FTC), and 19 (23.4%) lamivudine (LMV) (Table 1).

Twenty patients were positive for anti-HCV. Among them, 16 showed a detectable serum HCV-RNA (Table 1).

### 3.2. Characterization of HBV Replicative Activity by HBV Molecular Markers

By applying a highly sensitive ddPCR, characterized by a LLOQ of 1 IU/mL, cryptic serum HBV-DNA was detected in 29.6% (24/81) of patients with a median (IQR) value of 4 (1–15) IU/mL (Table 2), indicating low-level HBV replication in a conspicuous proportion of anti-HBc-positive HIV-infected patients despite HBsAg-negativity and the use of a cART containing > 1 anti-HBV drug.

Furthermore, three (3.6%) patients had detectable serum HBV-RNA below the lower limit of quantification: 1.65 log U/mL (Table 2), which is a result that supports a possible ongoing intrahepatic viral activity in these patients.

### 3.3. Factors Correlated with the Detection of Cryptic Serum HBV-DNA and HBV-RNA 

The next step of our study was to investigate parameters correlated with the detection of cryptic serum HBV-DNA and serum HBV-RNA.

Considering HIV characteristics at HIV diagnosis, cryptic serum HBV-DNA was significantly correlated with an advanced CDC stage. Indeed, 82.6% with cryptic serum HBV-DNA versus 52.6% without cryptic serum HBV-DNA have been diagnosed in the B or C stage (*p* = 0.01), supporting a more common condition of cryptic replication in patients receiving HIV diagnosis at a more advanced phase of HIV infection (Table 3).

Furthermore, considering HIV characteristics at blood sample collection, cryptic serum HBV-DNA tended to be more frequently revealed in patients with a detectable plasma HIV-RNA compared to patients with undetectable HIV-RNA (69.6% versus 47.4%, *p* = 0.07) (Table 3). In line with this result, a positive correlation was observed between cryptic serum HBV-DNA and plasma HIV-RNA (Rho:0.26, *p* = 0.02).

Considering HBV characteristics at blood sample collection, cryptic serum HBV-DNA was associated with anti-HBs titer <50 mIU/mL (58.3% of patients with cryptic serum HBV DNA had an anti-HBs titer <50 mIU/mL vs 36.8% of patients without cryptic viremia, *p* = 0.03) (Table 3).

Finally, cryptic serum HBV-DNA was more frequently detected in HIV-infected patients with chronic HCV infection (8 (33.3%) with versus 8 (14%) without cryptic serum HBV-DNA, *p* = 0.05) (Table 3).

No other parameters were significantly associated with the detection of cryptic serum HBV-DNA, including the type of anti-HBV drugs. Indeed, the percentage of patients with cryptic serum HBV-DNA was comparable in the three treatment groups: 27% for TAF (tenofovir alafenamide), 28% for TDF (tenofovir disoproxil fumarate), and 37% for LMV (lamivudine) (*p* = 0.7). 

Focusing on serum HBV-RNA, the positivity to this biomarker was correlated with a lower nadir CD4+ T cell count (3/3 (100%) patients with positive HBV-RNA versus 22/74 (29.7%) patients with negative HBV-RNA, which had a nadir CD4+ T cell count <100 cells/uL, *p* = 0.01).

### 3.4. Serological HBV Markers Predictive of Cryptic Serum HBV-DNA

An anti-HBs titer is known to reflect the extent of immunological control, while anti-HBc titer was recently proposed to predict the presence of a transcriptionally active intra-hepatic reservoir [25]. Thus, we investigated the role of these biomarkers in predicting cryptic HBV replication.

Interestingly, by AUROC, the combination of anti-HBs titer < 50 mIU/mL plus anti-HBc > 15 COI predicted the presence of cryptic HBV viremia with the best diagnostic accuracy (72.8%), which is a positive predictive value of 62.5% and a negative predictive value of 72% (Table 4). Indeed, 63% of patients with anti-HBs < 50 IU/mL plus anti-HBc > 15 COI had a cryptic serum HBV-DNA compared to 26% of those without this combination (OR (95%CI): 4.7 (1.1–21.7), *p* = 0.046).

## 4. Discussion

By using highly-sensitive molecular assays, this study provides evidence of a cryptic HBV replicative activity (not detected by the classical quantitative PCR assays) in a conspicuous proportion of anti-HBc-positive HIV-infected patients, despite HBsAg-negativity and the use of an HBV-active cART. This cryptic HBV replication may be predicted by the integrated use of anti-HBc and anti-HBs titers, suggesting the added value of novel HBV biomarkers in managing HIV-infected patients with a serological profile compatible with occult HBV infection. 

In particular, around 30% of anti-HBc-positive/HBsAg-negative HIV-infected patients has a cryptic serum HBV-DNA by ddPCR despite pharmacological pressure (median value of 4 (1–15) IU/mL). This result is in line with a previous study, led in South Africa, showing a persistently detectable serum HBV-DNA in 10/14 anti-HBc-positive/HBsAg-negative HIV-infected patients despite an HBV-active cART [27]. It is known that, in anti-HBc-positive/HBsAg-negative patients, anti-HBV immune responses play a key role in controlling cccDNA transcriptional activity and, in turn, in promoting cccDNA silencing [12]. It is conceivable that the HIV-driven weakening of immune responses can dysregulate this equilibrium, thus inducing low levels of HBV replication [9,28]. Notably, in line with this concept, we found an association between crytpic serum HBV-DNA and an advanced CDC stage at HIV diagnosis. Furthermore, we observed that cryptic serum HBV-DNA was significantly associated with an anti-HBs titer < 50mIU/mL, reflecting suboptimal anti-HBV immune responses. This result extends previous findings, showing a higher prevalence of cryptic serum HBV-DNA in patients with isolated anti-HBc [8,9,29]. Although still controversial, this finding supports the need to deepen the issue of anti-HBV vaccination in anti-HBc-positive patients with HIV infection, which is, so far, not yet recommended by guidelines promoted by the European AIDS Clinical Society (EACS) due to the lack of robust data [3,7,30].

Beyond cryptic serum HBV-DNA, three HIV-infected patients were positive for serum HBV-RNA, which is a new biomarker that has been proposed to reflect cccDNA transcriptional activity [31]. Notably, we found that the detection of serum HBV-RNA was significantly associated with a lower nadir CD4+ T cell count, again supporting the role of anti-HBV immune responses in controlling the transcriptional activity of an intrahepatic HBV reservoir. 

Overall findings support the importance of highly sensitive biomarkers to detect HBV replicative activity in the setting of anti-HBc-positive/HBsAg-negative HIV-infected patients. This is in line with the current EACS guidelines highlighting the importance to detect cryptic viremia in anti-HBc-positive/HBsAg-negative HIV-infected patients [7]. 

There is increasing evidence that anti-HBc-positive/HBsAg-negative patients (with or without HIV infection) are characterized by an increased progression of hepatic fibrosis, end-stage liver diseases, and at risk of HBV reactivation under severe immunosuppression [3,12,32,33,34,35]. In this light, it is conceivable that cryptic serum HBV-DNA may represent an important co-factor contributing to faster liver disease progression in the setting of anti-HBc-positive/HBsAg-negative HIV-infected patients. Further longitudinal studies on a large sample size are necessary to elucidate this issue.

The detection of cryptic HBV replication also has implications for an HIV-infected patient candidate to TDF withdrawal. So far, there is no clear indication on the possibility to suspend TDF in anti-HBc-positive/HBsAg-negative HIV-infected patients [7]. Nevertheless, a previous study reported two clinical cases of anti-HBc-positive/HBsAg-negative HIV-infected patients experiencing a re-uptake of HBV replication as a consequence of TDF withdrawal for simplification therapy [36]. Similarly, another study showed that 10.0% (6/60) of anti-HBc-positive patients developed HBV reactivation following TDF suspension [37]. In this light, the detection of cryptic HBV replication could help identify patients who need to continue an HBV-active cART since they are more prone to HBV-reactivation following TDF withdrawal. Further studies are necessary to unravel this issue critical for an optimal tailoring of treatment for HIV-infected patients.

Notably, we found that cryptic serum HBV-DNA tended to be more frequently detected in patients with detectable plasma HIV-RNA. In line with this result, a significant positive correlation between cryptic serum HBV-DNA and plasma HIV-RNA was observed. These findings are in keeping with a recent study showing worse HIV control in anti-HBc-positive HIV-infected patients than in HIV mono-infected patients and a correlation between the status of anti-HBc positivity and an HIV virological rebound [6]. The role of coinfections in exacerbating HIV replication and disease progression is known [38]. Although a causal link cannot be directly established, this raises the intriguing hypothesis of a potential HIV-HBV interaction that deserves further investigation.

Finally, in line with previous studies, an association between cryptic serum HBV-DNA and chronic HCV infection was observed mainly attributable to the shared transmission route of these two viruses [10,15].

It should be considered that the quantification of cryptic serum HBV-DNA does not allow us to identify anti-HBc-positive/HBsAg-negative individuals with transcriptionally inactive cccDNA. Like other DNA viruses, these represent the individuals in which HBV has established a virological latency, characterized by no viral particles production usually associated with limited clinical manifestations. Nevertheless, an adequate monitoring of serum HBV-DNA by highly-sensitive assays could be useful to identify patients breaking virological latency and, thus, are at higher risk of viral reactivation and disease progression, which is a critical aspect particularly in the setting of HIV-driven immunocompromise.

This is one of the first studies aimed at evaluating anti-HBc titers in the setting of HIV infection, particularly in those with a serological profile compatible with occult HBV infection. In our study, anti-HBc-positive/HBsAg-negative HIV-infected patients were characterized by a median (IQR) anti-HBc titer of 4.2 (2.4–11.6) COI. A recent study, led in liver biopsies from anti-HBc-positive/HBsAg-negative patients (without HIV-infection), showed that an anti-HBc IgG value >4.4 COI can predict the presence of intrahepatic cccDNA [25], supporting the role of this biomarker in reflecting the intrahepatic HBV reservoir. Notably, our study extended this concept by supporting the integrated use of anti-HBc and anti-HBs in identifying patients with cryptic HBV replication despite HBsAg-negativity. In particular, we found that an anti-HBs <50 mIU/mL (reflecting a lower anti-HBV immune responses) combined with an anti-HBc >15 COI (reflecting HBV replicative potential) can help predict cryptic serum HBV-DNA with a valuable diagnostic accuracy. This result supports the integrated use of accurate serological markers in the management of anti-HBc-positive/HBsAg-negative patients with HIV infection. Further studies, led on a larger sample size, are necessary to validate this finding.

In conclusion, cryptic HBV replication (not detected by classical assays) characterizes a conspicuous set of anti-HBc-positive HIV-infected patients despite HBsAg-negativity and antiviral-treatment. The integration of serological and virological markers may help identify patients with cryptic HBV replication, thus optimizing the monitoring of anti-HBc-positive/HBsAg-negative patients.

## Figures and Tables

**Table 1:** Demographic, clinical, and virological characteristics of the study population.

Patients’ Characteristics (N = 81)	
Sex Male, N (%)	58 (71.6)
Median (IQR) Age, years	55.2 (47.3–63.1)
**Risk factor, N (%)**	
Homosexual	32 (39.5)
Heterosexual	26 (32.1)
Bisexual	23 (28.4)
**Ethnicity, N (%)**	
Caucasian	70 (86.4)
African	8 (9.9)
Other	3 (3.7)
**Characteristics of HIV infection at HIV diagnosis**	
Median (IQR) CD4 cell count, cells/uL	155 (68–317)
Median (IQR) plasma HIV-RNA, copies/mL	111,995 (49,957–404,944)
CDC criteria, N (%) ^b^	
A	31 (38.8)
B	21 (26.2)
C	28 (35.0)
**Characteristics of HIV infection at sample collection ^a^**	
Median (IQR) CD4 cell count, cells/uL	541 (349–716)
Median (IQR) plasma HIV-RNA, copies/mL	<20 (<20–38)
HIV-RNA < 20 copies/mL, N (%)	60 (75.0)
**Liver functionality tests**	
Median (IQR) ALT, U/L	26 (21–37)
Median (IQR) AST, U/L	26 (22–33)
**HBV serological markers**	
HBsAg negative/Anti-HBc positive, N (%)	81 (100)
Anti-HBe positive, N (%)	18 (34.6) ^c^
Anti-HBs positive, N (%)	58 (71.6)
Anti-HBs positive, Median (IQR), mIU/mL	277 (90–957)
**Therapy, N (%)**	
TAF/FTC ^d^ containing antiretroviral therapy	37 (45.7)
TDF/FTC ^e^ containing antiretroviral therapy	25 (30.9)
LMV ^f^ containing antiretroviral therapy	19 (23.4)
Therapy Duration, Median (IQR) months	35 (17–61)
**HCV coinfection, N (%)**	
Anti-HCV Positive	20 (24.7)
Anti-HCV positive, HCV-RNA Positive	16 (19.8)

^a^ HIV-RNA at sample collection was available for 80/81 patients. Thus, the percentage of patients in the different categories of HIV viremia was calculated on 80 patients with available data. ^b^ The CDC stage was available for 80/81 patients. Thus, the percentages of patients in the different CDC stages was calculated on 80 patients. ^c^ The datum is calculated on 52/81 patients with an available Anti-HBe result. ^d^ TAF/FTC: tenofovir alafenamide + emtricitabine. ^e^ TDF/FTC: Tenofovir disproxil fumarate + emtricitabine. ^f^ LMV: lamivudine.

**Table 2:** Results of novel serological and molecular HBV markers in the study population.

Innovative HBV Marker Results	N Patients	Value
Highly Sensitive HBsAg detection ^a^	81 (100%)	Negative
Quantitative anti-HBc, Median (IQR) COI	81 (100%)	4.2 (2.4–11.6)
Serum HBV-DNA Positive by ddPCR, Median (IQR) IU/mL	24 (29.6)	4 (1–15)
Serum HBV-RNA Positive, Levels, IU/mL	3 (3.6)	Detected (<1.65 log) ^b^

Abbreviations: ddPCR, digital droplet PCR ^a^ HBsAg was detected by the highly sensitive Abbott ARCHITECT HBsAg NEXT Qualitative assay (HBsAgNx). ^b^ 1.65 log IU/mL represents the limit of quantification of the assay for serum HBV-RNA quantification.

**Table 3:** Factors correlated with detection of HBV cryptic viremia.

Patients’ Characteristics	HBV-DNA Positive by ddPCR	HBV-DNA Negative by ddPCR	*p*-Value ^c^
	(*n* = 24)	(*n* = 57)	
**Male, N (%)**	19 (79.2)	43 (75.4)	0.71
**Origin, N (%)**			
Caucasian	22 (91.7)	48 (84.2)	0.79
African	1 (4.2)	7 (12.3)	
Other	1 (4.2)	2 (3.5)	
**Characteristics at HIV diagnosis**			
Plasma HIV-RNA, copies/mL	236,731 (146,476–762,639)	89,825 (46,656–331,167)	0.11
CD4+ cell count, cells/mm^3^	120 (50–249)	178 (80–332)	0.33
**CDC stage, N (%) ^a^**			
A	4 (17.4)	27 (47.4)	**0.01**
B and C	19 (82.6)	30 (52.6)	
**HIV characteristics at sample collection**			
Detectable plasma HIV-RNA, N (%) ^b^	16 (69.6)	27 (47.4)	0.07
CD4+ cell count, cells/mm^3^	426 (331–701)	552 (379–735)	0.58
**Liver functionality tests at sample collection**			
Median (IQR) ALT, U/L	24 (21–29)	26 (20–38)	0.54
Median (IQR) AST, U/L	26 (22–33)	26 (20–38)	0.79
**HBV characteristics at sample collection**			
Anti-HBs negative < 50 mIU/mL	15 (58.3)	21 (36.8)	**0.03**
Median (IQR) Anti-HBs titer, mIU/mL	60 (36–470)	361 (139–1000)	**0.05**
Mean (min-max) Anti-HBc titer, COI	17 (1–58)	8 (1–29)	**0.04**
**HCV coinfection at sample collection**			
Anti-HCV Positive, HCV-RNA positive	8 (33.3)	8 (14.0)	**0.05**

^a^ Datum available for 80/81 patients: 23/24 with HBV cryptic viremia and 57/57 without HBV cryptic viremia. ^b^ Detectable HIV-RNA indicates HIV-RNA detected below the limit of quantification and ≥20 copies/mL. This datum was available for 80/81 patients (23/24 with HBV cryptic viremia and 57/57 without HBV cryptic) and the relative percentages have been calculated accordingly. ^c^ Statistically significant *P* Values are reported in bold.

**Table 4:** Diagnostic performance of serological HBV markers in predicting cryptic serum HBV-DNA.

Cut-offs of HBV Markers	PPV	NPV	Accuracy
Anti-HBc > 15 COI	50%	73.9%	70.4%
Anti-HBs < 50 mIU/mL	41.7%	80%	63%
Anti-HBs < 50 mIU/Ml + Anti-HBc > 15 COI	62.5%	72%	72.8%

Abbreviations: Anti-HBc, Hepatitis B core antibodies. COI, cut-off index. Anti-HBs, Hepatitis B surface antibodies.
